# Effectiveness of Standardized Nursing Care Plans in Health Outcomes in Patients with Type 2 Diabetes Mellitus: A Two-Year Prospective Follow-Up Study

**DOI:** 10.1371/journal.pone.0043870

**Published:** 2012-08-27

**Authors:** Juan Cárdenas-Valladolid, Miguel A. Salinero-Fort, Paloma Gómez-Campelo, Carmen de Burgos-Lunar, Juan C. Abánades-Herranz, Rosa Arnal-Selfa, Ana López- Andrés

**Affiliations:** 1 Unidad de Apoyo Técnico, Gerencia Adjunta de Planificación y Calidad, Servicio Madrileño de Salud, Madrid, España; 2 Fundación Investigación Biomédica, Hospital Carlos III, Madrid, España; 3 Unidad de Epidemiología Clínica, Hospital Carlos III, Madrid, España; 4 Dirección Técnica de Formación e Investigación, Gerencia Adjunta de Planificación y Calidad, Servicio Madrileño de Salud, Madrid, España; 5 Dirección de enfermería, Dirección Asistencial Norte, Gerencia Adjunta Asistencial, Servicio Madrileño de Salud, Madrid, España; 6 Departamento de Medicina Preventiva y Salud Pública, Facultad de Ciencias de la Salud, Universidad Rey Juan Carlos, Madrid, España; McGill University, Canada

## Abstract

**Background:**

Implementation of a standardized language in Nursing Care Plans (SNCP) allows for increased efficiency in nursing data management. However, the potential relationship with patientś health outcomes remains uncertain. The aim of this study was to evaluate the effectiveness of SNCP implementation, based on North American Nursing Diagnosis Association (NANDA) and Nursing Interventions Classification (NIC), in the improvement of metabolic, weight, and blood pressure control of Type 2 Diabetes Mellitus (T2DM) patients.

**Methods:**

A two-year prospective follow-up study, in routine clinical practice conditions. 31 primary health care centers (Spain) participated with 24,124 T2DM outpatients. Data was collected from Computerized Clinical Records; SNCP were identified using NANDA and NIC taxonomies. Descriptive and ANCOVA analyses were conducted.

**Results:**

18,320 patients were identified in the Usual Nursing Care (UNC) group and 5,168 in the SNCP group. At the two-year follow-up, the SNCP group improved all parameters except LDL cholesterol and diastolic blood pressure. We analyzed data adjustming by the baseline value for these variables and variables with statistically significant differences between groups at baseline visit. Results indicated a lowering of all parameters except HbA1c, but a statistically significant reduction was only observed with diastolic blood pressure results. However, the adjusted reduction of diastolic blood pressure is of little clinical relevance. Greater differences of control values for diastolic blood pressure, HbA1c, LDL-cholesterol and Body Mass Index were found in the SNCP group, but only reached statistical significance for HbA1c. A greater proportion of patients with baseline HbA1c ≥7 decreased to <7% at the two-year follow-up in the SNCP group than in the UNC group (16.9% vs. 15%; respectively; p = 0.01).

**Conclusions:**

Utilization of SNCP was helpful in achieving glycemic control targets in poorly controlled patients with T2DM (HbA1c ≥7%). Diastolic blood pressure results were slightly improved in the SNCP group compared to the UNC group.

**Trial Registration:**

ClinicalTrials.gov NCT01482481

## Introduction

Type 2 Diabetes Mellitus (T2DM) is a chronic disease that has increased its prevalence and incidence rates in recent years [1], and some authors consider it the most important epidemic of the 21^st^ century [2]. It is also associated with premature morbidity and mortality [3], [4] as well as with an increase in healthcare costs [5].

Glycated hemoglobin (HbA1c) is an important indicator of diabetic control, because it provides an average of all the blood glucose readings for the previous two-three months [6]. Several studies [7]–[9] have shown a relationship between the lack of glycemic control (HbA1c>7%) and chronic complications, so the relative risk for stroke or coronary heart disease is 1.18 for each 1-percent point increase in HbA1c (95% Confidence Interval [95% CI] = 1.10–1.26) in patients with T2DM [10].

Currently, the responsibility for the care of patients with diabetes has shifted to a primary health care setting, and, more specifically, to nurses. They have a central role in the treatment of patients with T2DM and have been implementing a wide range of interventions aimed at improving the provision of diabetes care and achieving better metabolic control [11].

**Figure 1:**
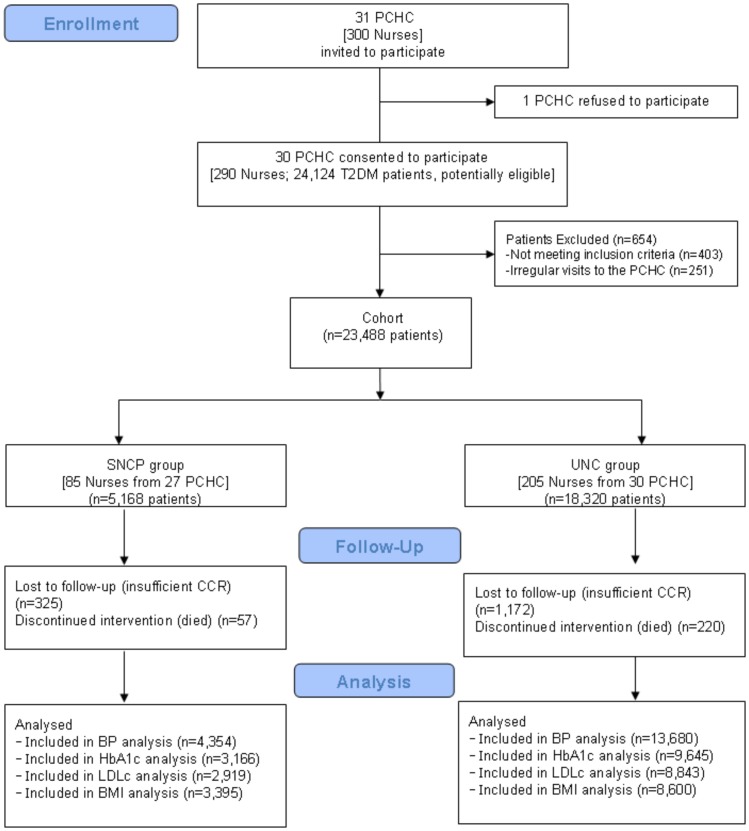
Flow diagram of participants. PCHC: Primary Health Care Center; T2DM: Type 2 Diabetes Mellitus; SNCP: Standardized Nursing Care Plans; UNC: Usual Nursing Care; CCR: Computerized Clinical Records; BP: Blood Pressure; HbA1c: Glycated hemoglobin; LDLc: Low-density lipoprotein cholesterol; BMI: Body Mass Index.

In the last decade, there has been enormous development in the implementation of standardized languages in Nursing Care Plans (SNCP) for nursing diagnoses (North American Nursing Diagnosis Association -NANDA) [12] and interventions (Nursing Interventions Classification -NIC) [13]. In Spain since 1998, these taxonomies have been progressively incorporated into normal clinical practice and Computerized Clinical Records (CCR). However, there is still no common language in Spanish nursing practice [14].

The implementation of SNCP care plans allow for increased practicality and efficiency in nursing data management [15], but the potential relationship between nursing interventions and patientś health outcomes remains uncertain [16], [17].

The aim of the study was to evaluate the effectiveness of implementing SNCP in CCR registration in the improvement of metabolic, weight, and blood pressure control of patients with T2DM after two–year follow-up. The SNCP followed NANDA and NIC taxonomies.

**Table 1:** Nursing diagnoses: domains, class, and titles.

Domain	Class	Nursing diagnoses	
Nutrition	Ingestion	00001	Imbalanced nutrition: more than body requirements
		00002	Imbalanced nutrition: less than body requirements
		00003	Risk for imbalanced nutrition: more than body requirements
Coping/stress tolerance	Coping responses	00069	Ineffective coping
Life Principles	Value/Belief/Action Congruence	00079	Non compliance
Health promotion	Health management	00078	Ineffective self health management
		00080	Ineffective family therapeutic Regimen Mangament
		00081	Ineffective therapeutic regimen management
		00082	Effective therapeutic regimen management
		00162	Readiness for enhanced self-health management
		00163	Readiness for enhanced nutrition
		00084	Health-Seeking Behaviors: Management DM
Self Perception	Self-Esteem	00120	Situational Low self-esteem
Perception/cognition	Cognition	00126	Deficient knowledge
Activity/Rest	Activity/Exercise	00168	Sedentary Lifestyle
Safety/Protection	Physical injury	00046	Impaired skin integrity

**Table 2:** Nursing interventions: domains, class, and titles.

Domain	Class	Nursing interventions	
Physiological: Basic and Complex	Activity and exercise management	0200	Exercise promotion
		1020	Diet Staging
		1030	Eating Disorders: Management
		1100	Nutrition Management
		1160	Nutritional Monitoring
		1240	Weight gain assistance
		1260	Weight Management
		1280	Weight Reduction Assistance
		5246	Nutritional Counseling
	Self-Care Facilitation	1803	Self-Care Assistance: Feeding
	Electrolyte and Acid-Base Management	2120	Hyperglycemia management
		2130	Hypoglycemia management
Behavioral	Behavioral Therapy	4360	Behavior Modification
		4410	Mutual Goal Setting
		4420	Patient Contracting
		4470	Self-Modification Assistance
	Cognitive Therapy	4700	Cognitive Restructuring
	Communication Enhancement	4920	Active Listening
	Coping assistance	5210	Anticipatory Guidance
		5220	Body Image Enhancement
		5230	Coping Enhancement
		5240	Counseling
		5250	Decision-Making Support
		5270	Emotional Support
		5330	Mood Management
		5390	Self-Awareness Enhancement
		5400	Self-Esteem Enhancement
		5440	Support System Enhancement
		5480	Values Clarification
	Patient education	5510	Health Education
		5520	Learning Facilitation
		5540	Learning Readiness Enhancement
		5602	Teaching: disease process
		5606	Teaching: individual
		5612	Teaching: prescribed activity/exercise
		5614	Teaching: prescribed diet
		5616	Teaching: prescribed medication
		5618	Teaching: procedure/treatment
		5620	Teaching: psychomotor skills
Safety	Crisis Management	6160	Crisis Intervention
	Risk management	6520	Health Screening
		6610	Risk Identification
		6650	Surveillance
		6680	Vital sings monitoring
	Lifespan care	7140	Family Support
Health System	Delivery System	7400	Health System Guidance
		7460	Patient Rights Protection
	Information Management	8180	Telephone Consultation
		8190	Telephone Follow-up

## Methods

### Design

A two-year prospective follow-up study, carried out during the period from March 2008 to February 2010.

### Sample

24,124 T2DM patients were potentially eligible to be included in the study. These patients were identified using the CCR and were comprised of patients who regularly visit (at least two records in the CCR over the past year) the 31 primary health care centers in the northeastern urban area of Madrid, Spain.

Eligibility criteria for patients were: over 30 years of age, with previously diagnosed T2DM (cardinal clinical, plus random blood glucose >200 mg/dl or oral glucose of >200 mg/dl at 2 h, twice or plasma fasting glucose of >126 mg/dl on two occasions or previously diagnosed). Patients were not included if they met any of the following exclusion criteria: gestational diabetes, patients involved in clinical trials, patients with life expectancy of less than one year (according to clinical judgment), and homebound patients. Figure 1 show the flowchart of the study.

During February 2006- February 2008, the vast majority of nurses in the primary health care centers were trained in diagnostic reasoning based on NANDA and NIC taxonomies. Training consisted of eight classes of two hours, taught by a specialized nurse. 94.12% of nurses in the SNCP group and 24.39% of nurses in the UNC group attended the training. The implementation of NANDA and NIC taxonomies in the CCR by primary health care centers in Madrid began in March 2008.

Based on the types of nursing actions registered in the CCR, two groups of nurses were identified: those that used usual nursing care (UNC) and those that used SNCP.

The first group applied UNC which is defined as: direct nursing care, non standardized clinical interventions that contribute to the health or recovery of a patient. UNC for patients with T2DM are defined as: the treatment and monitoring of T2DM including interventions at different levels such as: controlling blood sugar levels, control of cardiovascular risk factors, drug therapy compliance, change in lifestyles, health education, and self-management [18]. UNC were identified in the CCR based on the non-standardized languages in nursing care or the standardized nursing cares based on other taxonomies.

**Table 3:** Baseline characteristics of the study population.

	UNC (n: 18,320)	SNCP (n: 5,168)	p value
**Sociodemographic variables**			
Female gender (%)	51.5	52.3	0.299
Age (yr) [mean ±SD]	69±12	70±13	0.000
**Clinical variables**			
Diabetes evolution time (yr) [mean ±SD]	7.4±6.3	8.6±6.9	0.000
**Personal health habits**			
Smoking (%)	19.4	18.7	0.268
Drinking (%)	20.9	23.4	0.000
Physical activity (sedentary) (%)	1.8	4.1	0.000
**Biological parameters**			
BMI (Kg/m^2^) [mean ±SD]	30.1±5	29.8±4.7	0.004
**DM treatment profile** %			
Drug-free	11.3	8	0.000
Oral antidiabetic	61.5	69.8	0.000
Insulin	18.6	22.6	0.000
Oral antidiabetic + Insulin	9.6	13.4	0.000
**Others treatments** %			
Statins	47.7	52.3	0.000
Fibrates	3.7	3.5	0.585
Diuretics	27.9	26.5	0.054
Beta-blockers	16.7	15.6	0.066
Calcium antagonist	21.1	22.5	0.031
ACE Inhibitors	36.2	37.3	0.135
ARB	22.6	25.2	0.000
Antiplatelet	58.0	62.7	0.000
**Associated morbidity** %			
CHD	13.3	13.2	0.949
Dyslipidemia	44.8	49.2	0.000
Hypertension	69.6	68.6	0.160
**Complications** %			
Retinopathy	3.5	4.5	0.001
Nephropathy	6.8	7.1	0.351
Neuropathy	1.8	1.7	0.947

UNC: Usual Nursing Cares; SNCP: Standardized Nursing Care Plans; DM: Diabetes mellitus; BMI: Body mass index; ACE: Angiotensin-converting enzyme; ARB: Angiotensin receptor blockers; CHD: Coronary heart disease.

The second group, SNCP group, applied UNC for patients with T2DM and SNCP based on NANDA and NIC taxonomieś. A SNCP describes the care to be provided to a specific group of patients and contains a diagnostic statement, nursing goals, implementation and evaluation [19]; based on up-to-date, evidence-based knowledge.

In the CCR, SNCP was identified based on the following three criteria:

- Criterion 1. The patient has a code that corresponds to the Gordon’s functional health patterns [20] in at least one of the following areas: health perception and health management; nutritional and metabolic; and activity and exercise.

- Criterion 2. The problems identified were described using codes nursing diagnosis statements based on NANDA taxonomy, used in T2DM patients (Table 1). A nursing diagnosis based on NANDA taxonomy is defined as a clinical judgment about individual, family or community responses to actual or potential health problems or life processes which provide the basis for the selection of nursing interventions to achieve patientś outcomes, for which, the nurse is accountable [12].

- Criterion 3. The nursing intervention carried out was registered according to the codes of NIC taxonomy, used in T2DM patients [13] (Table 2).

**Table 4:** Mean values (SD) and changes of baseline and final parameters in both groups.

	SNCP	UNC	Unadjusted SNCP effect (95% CI)	Adjusted SNCP effect(95% CI)
**Hba1c** (%) *mean* (SD)				
N patients	3,166	9,645		
Baseline	7.25 (1.2)	7.12 (1.2)		
Final	7.02 (1.1)	6.92 (1.1)		
Change	−0.23 (1.1)	−0.20 (1.1)	−0.02 (0.02–0.07)	0.03 (−0.01–0.06)
p value			0.26	0.14
**LDL Cholesterol** (mg/dl) *mean* (SD)				
N patients	2,919	8,843		
Baseline	115 (31)	119 (33)		
Final	108 (30)	111 (31)		
Change	−7.14 (30)	−8.04 (32)	0.90 (2.22–0.42)	−0.68 (−1.76–0.39)
p value			0.18	0.21
**SBP** (mmHg) *mean* (SD)				
N patients	4,354	13,680		
Baseline	134 (16)	134 (17)		
Final	132 (15)	132 (15)		
Change	−1.95 (19)	−1.48 (18)	−0.46 (−1.08–0.16)	−0.07 (−0.56–0.42)
p value			0.15	0.78
**DBP** (mmHg) *mean* (SD)				
N patients	4,354	13,680		
Baseline	76 (10)	76 (10)		
Final	75 (9)	74 (9)		
Change	−1.45 (11)	−1.46 (11)	0.01 (0.37–0.36)	−0.33 (−0.63–0.04)
p value			0.98	0.02
**BMI** (Kg/m^2^) *mean* (SD)				
N patients	3,395	8,600		
Baseline	29.8 (4.7)	30.1 (5)		
Final	29.5 (4.7)	29.8 (5)		
Change	−0.22 (1.9)	−0.22(1.8)	−0.00 (−0.08–0.07)	−0.02 (−0.10–0.05)
p value			0.99	0.54

UNC: Usual Nursing Cares; SNCP: Standardized Nursing Care Plans; Hba1c: Glycated hemoglobin; LDL: Low-density lipoprotein; SBP: Systolic Blood Pressure; DBP: Diastolic blood pressure; BMI: Body mass index.

### Sources of Information

The CCR for primary health care in Madrid’s Health Service was used as the data source and was administered by OMI-AP® software. The CCR has been previously validated [21].

Data extraction of patient information was conducted at four time points: baseline, 12 and 18 months and after complete follow-up of the study (two-year follow-up). Data included variables and date on which data had been recorded in the CCR. The collection of variables was performed in routine clinical practice conditions.

**Table 5:** Percentage of subjects on-target for cardiovascular risk factors at baseline and at the two-year follow-up, stratified by group (UNC/SNCP).

	N	Baseline (%)	12 Months (%)	18 Months (%)	24 Months (%)	p-value*	Change (%)	p-value
**SBP**<130 mmHg								
UNC (%)	13,680	31.6	33.5	35.6	35.5	<0.01	3.9	<0.01
SNCP (%)	4,354	31.6	32.9	35.5	34.5	<0.01	2.9	
**DBP**<80 mmHg								
UNC (%)	13,680	50.6	53	55.7	55.9	<0.01	5.3	0.31
SNCP (%)	4,354	53.7	57	59.7	59.4	<0.01	5.7	
**HbA1c** <7%								
UNC (%)	9,645	54.4	59.2	61.2	60.3	<0.01	5.9	<0.01
SNCP (%)	3,166	47.6	51.8	56.4	55.2	<0.01	7.6	
**LDL c** <130 mg/dl								
UNC (%)	8,843	29.8	32.3	36.1	38.0	<0.01	8.2	0.61
SNCP (%)	2,919	33.4	34.9	38.2	41.9	<0.01	8.5	
**BMI** <30 Kg/m^2^								
UNC (%)	8,600	54.2	54.8	56.3	56.6	<0.01	2.4	0.21
SNCP (%)	3,395	56.8	58.2	59.6	59.6	<0.01	2.8	

p*: p value of comparison of the two-year follow-up and baseline values, intragroup differences; Change: Final value (two-years)– Baseline values; UNC: Usual Nursing Cares; SNCP: Standardized Nursing Care Plans; SBP: Systolic Blood Pressure; DBP: Diastolic blood pressure. HbA1c: Glycated hemoglobin; LDL c: Low-density lipoprotein cholesterol; BMI: Body mass index.

For all the patients, the following variables were recorded: sociodemographic (age, gender), clinical variables (diabetes evolution time), personal health habits (smoking: cigarettes/day; drinking: alcohol units/week; physical activity: measured in hours per week with any exercise or activity outside of the patients’ regular job being considered, and recoded as sedentary, moderate-intensity, vigorous-intensity), associated morbidity (hypertension, dyslipidemia, coronary heart disease-CHD), diabetes mellitus complications (retinopathy, nephropathy, neuropathy), and the type of treatment prescribed (pharmacological and dietary). Additionally, biochemical–biological parameters were collected: body mass index (BMI), systolic blood pressure (SBP), diastolic blood pressure (DBP), total cholesterol, high-density lipoprotein (HDL) cholesterol, low-density lipoprotein (LDL) cholesterol, triglycerides, and HbA1c. Only patients with laboratory values and anthropometric records in the CCR at baseline and at final visit were included to determine the effect of SNCP and achievement of control objectives. In some cases, loss of data was close to 50% (LDL cholesterol).

Blood pressure was measured according to the recommendations of the *Seventh Report of the Joint National Committee on Prevention, Detection, Evaluation, and Treatment of High Blood Pressure* (2003) [22]; these recommendations were the most current at the start of this study.

The primary outcomes were those related to diabetes targets: glycemic control (HbA1c<7), blood pressure control (SBP<130 mm Hg, DBP<80 mmHg), lipid control (LDL cholesterol <130 mg/dl), and weight control (BMI<30 kg/m^2^) after the two-year follow-up.

The study was approved by the Institutional Review Board of the Committee on Human Research of the *Hospital Ramón y Cajal* (Madrid). The Committee for the Protection of Human Subjects determined that no informed consent was necessary in this type of study.

**Table 6:** Associated factors with HbA1c <7% in 12,800 DM patients with baseline HbA1c≥7%: logistic regression model.

Variables	Adjusted OR	95% CI	p value
**Group** (SNCP/UNC)	1,11	0,99–1,24	0,06
**Insulin treatment** (yes/no)	1,20	1,06–1,36	<0,01
**Oral antidiabetic agents** (yes/no)	2,41	2,12–2,75	<0,01
**Gender** (male/female)	1,16	1,05–1,28	<0,01
**Age**, yr	0,99	0,98–1	0,17

UNC: Usual Nursing Cares; SNCP: Standardized Nursing Care Plans; HbA1c: Glycated hemoglobin.

### Statistical Analysis

First, a descriptive analysis was carried out for each variable included in this study, involving the mean and standard deviation for the quantitative variables and frequencies measured for the qualitative variables. Student’s *t*-test, or its nonparametric equivalent, was used for paired data (Wilcoxon test). Furthermore, the Pearson χ^2^ test was used for qualitative variables, and McNemar’s test was used for paired data.

Change (mean at the two-year follow-up value − mean baseline value) was calculated in both groups for the following variables: LDL cholesterol, HbA1c, SBP, DBP, and BMI. The variable Effect of the SNCP was determined for these variables using the formula: mean value of the change in SNCP− mean value of the change in UNC. Covariance analysis methodology (ANCOVA) proposed by Vickers was used to determine the adjusted effect of SNCP [23]. The covariables (adjustment variables) were: the baseline value for these variables and variables with statistically significant differences between groups at baseline visits (age, drinking, physical activity – sedentary, BMI, and type of treatment) or clinical relevance (gender). In order to adjust for significantly different variables at baseline and between both groups multivariate techniques (ANCOVA, logistic regression) were performed in order to adjust by variables imbalanced at baseline values between both groups.

In all instances, the accepted level of significance was 0.05 or less, with 95% CI. All the analyses were carried out using the intention-to-treat principle. Statistical analysis of the data was carried out with SPSS 17.0 (SPSS, Inc., Chicago, Illinois, USA).

## Results

A total of 23,488 patients were included, of which 51.6% were female, mean age 69.7 years (SD = 14.5), and mean diabetes evolution time of 8.1 years (SD = 8.3). 18,320 patients were identified as part of the UNC group and 5,168 were identified as part of the SNCP group.

Table 3 show baseline characteristics of the study population. The two groups were homogeneous in gender, but not in age, and diabetes evolution time. Patients in the SNCP group had a higher prevalence of poorer personal health habits (drinking and sedentary physical activity), dyslipidemia and complications (retinopathy). Patients in the SNCP group also received more treatment for diabetes (oral antidiabetic and insulin) and for cardiovascular disease (ARB/calcium antagonist/statins/antiplatelets), with poorer HbA1c (7.25% vs. 7.12%; p<0.001), and better LDL cholesterol (115 mg/dl vs. 119 mg/dl; p<0.001), than patients of the UNC group.

At the two-year follow-up, both groups experienced a modest decline in their parameter values (Table 4). The unadjusted effect of SNCP improved health outcomes, except for with LDL cholesterol and DPB. After adjusting for baseline parameter values and age, sex, type of treatment and physical inactivity, a lowering effect on all health outcomes was observed except for HbA1c. A statistically significant reduction was only observed with DBP. However, the reduction of DBP was of little clinical relevance.

Table 5 shows the proportion of patients who achieved the target of glycemic, blood pressure, lipid and weight control, at baseline, 12, 18 and 24 months, in both groups. There was a significant improvement (p<0.01) in the percentage of subjects who complied with control targets in both groups, at the two-year follow-up. The SNCP group showed greater change in control values than the UNC group, in DBP, HbA1c, LDL cholesterol and BMI, but only reached statistical significance for HbA1c. The UNC group performed better than the SNCP group in the degree of control of SBP (p<0.01).

There was a greater proportion of patients with baseline HbA1c ≥7 who decreased this value below 7% at the two-year follow-up in the SNCP group that in the UNC group (16.9% vs. 15%, respectively; p<0.01).

Finally, table 6 shows the factors associated with achieving glycemic control in patients with baseline HbA1c ≥7%. After adjusting by type of treatment, age and gender, the SNCP group showed a favourable trend toward target control (OR = 1.11; 95%CI = 0.99–1.24; p: 0.06). The variable more strongly associated with glycemic control was oral antidiabetic agents (OR = 2.41; 95% CI = 2.12–2.75; p<0.01).

In the SNCP group, average use of NANDA taxonomy in nursing diagnoses was 6.4 (DS = 1.9) and the most frequently used nursing diagnoses included: Effective Therapeutic Regimen Management (33.9%); Ineffective Therapeutic Regimen Management (22.4%); Impaired Skin Integrity (12.3%); Health-Seeking behaviors (9.9%); Imbalanced Nutrition: more than Body Requirements (3.7%); Readiness for Enhanced Self Health Management (3.1%); Deficient knowledge (2.2%) and Non compliance (2%).

## Discussion

The present study showed that patients in the SNCP group reached a significant reduction in DBP, at the two-year follow-up, compared to patients in the UNC group. However, a reduction in DBP values has little clinical relevance. SNCP group demonstrated a favourable trend toward the glycemic control in previously poorly controlled patients, after adjusting for age, gender, and type of treatment. The main predictors variables were treatment with oral antidiabetic agents, and insulin treatment; that previously, in our country, had been associated with glycemic control [24].

Preceding studies have shown that the implementation of standardized languages in nursing care plans enhances the quality of documented patient assessments, the identification of commonly occurring diagnoses within similar settings, and coherence among nursing diagnoses, interventions and outcomes [17], [18], but that better documentation did not necessarily lead to better patient care outcomes [17].

Some studies in hospital settings, examined the relationship between the implementation of standardized languages and patient’s outcomes [25], [26]. However, there is a gap in the literature about the potential relationship between the implementation of standardized languages in nursing care plans and health outcomes for chronic patients in primary health care settings [16], [17]. One meta-analysis of nine trials that included 1,846 patients showed limited evidence that standardized electronic documentation of nursing diagnosis and related interventions led to better health outcomes [16].

The utilization of standardized languages in nursing care plans may be interpreted as an organizational intervention aimed at improving the process of care or patient outcomes. In a systematic review [27], which included nine studies of organizational interventions in patients with diabetes, there was no evaluation of the effectiveness of SNCP or nursing diagnoses. For this reason, our study cannot be compared with similar efficacy studies.

The patients in the SNCP group had a greater risk profile. This is consistent with the findings of Paans et al. [28] who identifies that one of the factors associated with the use of nursing diagnoses is the complexity of a patientś situation. For this reason, we adjusted for baseline differences with a multivariate analysis (ANCOVA), in spite of this there is still a possibility of bias in favor of the null hypothesis.

The study sample was composed of patients with T2DM who regularly visited primary health care centers. This may not be representative of the entire T2DM patient community. However, the prevalence of diabetes mellitus recorded in the 31 participating primary health care centers [21] is similar to that found in a population based study carried out in our city [29] (5.02% vs. 6.3%, respectively), so the potential selection bias would be of small magnitude.

Finally, the level of evidence from cohort studies is lower than clinical trials, so our results should be interpreted with caution.

Despite the limitations, this research analyzed a gap in the literature about the unclear relationship between the application of SNCP and patient outcomes. In conclusion, SNCP appears to be helpful in achieving target HbA1c levels in patients with T2DM with previously poorly controlled (HbA1c ≥7%). Other control parameters (blood pressure) are slightly improved compared to UNC. Clinical trials are needed to confirm our findings.
